# Associations of Homebound State in Older Adults With Neighborhood Social Cohesion

**DOI:** 10.1177/07334648251360520

**Published:** 2025-07-11

**Authors:** Namkee G. Choi, Brian Fons, Angelina Gutierrez, Kelly Vences

**Affiliations:** 1 12330 The University of Texas at Austin, Austin, TX, USA

**Keywords:** homebound, frequency of going outside, neighborhood, social cohesion, physical disorder, social network

## Abstract

Using data from the 2022-2023 U.S. National Health and Aging Trends Study, we examined the associations between neighborhood social cohesion (NSC) and homebound state (i.e., never/rarely went outside in the past month) in 2022 (*N* = 5,593), and between changes in NSC and changes in the homebound state between 2022 and 2023 (*N* = 4,907). In 2022, 4.9% of the study population were homebound, and higher perceived NSC was associated with lower odds of a homebound state (aOR = 0.79, 95% CI = 0.73-0.86, compared to 5+ times weekly outings), controlling for social support network size, neighborhood physical disorder, and sociodemographic and health characteristics. Between 2022 and 2023, 2.2% transitioned out of the homebound state, but 4.3% remained homebound or became newly homebound. Favorable changes in NSC were associated with higher odds (RRR = 1.63, 95% CI = 1.01-2.62) of recovery from homebound state the following year. These findings have important implications for considering interventions to increase social capital for older adults.

**What this paper adds**

• Higher perceived neighborhood social cohesion (NSC) is associated with lower odds of homebound state.
• Favorable changes in NSC were associated with a higher likelihood of transitioning from homebound to no longer homebound between 2022 and 2023. A smaller social support network was associated with a higher likelihood of continuing in or transitioning into a homebound state.
• Black and Hispanic older adults had greater odds of being homebound and remaining homebound, after controlling for other demographic and health status variables.

**Applications of study findings**

• It is important to consider interventions to increase social capital to better meet the health and social needs of the increasing number of homebound older adults.
• Community/neighborhood-based programs aimed at increasing social connectivity and activity engagement may facilitate homebound prevention and recovery.
• These programs are especially needed for Black and Hispanic homebound older adults, as they are more likely to remain homebound compared to their non-Hispanic White counterparts.

## Introduction

There is well-established evidence that individuals’ cognitive, physical/functional, and mental health are influenced by both economic and social capitals and the policies that shape them. Economic capital refers to individual and community-wide material conditions, while social capital refers to the actual and potential resources that are often rooted in social cohesion, that is, social networks and features such as interpersonal trust, norms of reciprocity, and social engagement that foster community and social participation, especially in the neighborhood or local area (Carpiano, 2006).

A considerable body of research has indeed shown that neighborhood social cohesion (NSC), assessed with perceived attachment, connectedness or belonging, reciprocity, and interpersonal trust, is a significant factor for cognitive, physical, and mental health, physical activity, other health-related behaviors, preventive healthcare use, disease outcomes, and mortality in older adults (Choi & Matz-Costa, 2018; Gullett et al., 2022; Kim et al., 2020; Kim & Kawachi, 2017; Matsuura et al., 2023; Quinn et al., 2019; Rosenblatt et al., 2021; Ruiz et al., 2019; Zaheed et al., 2019). NSC is a particularly important factor for health in older adults, as they tend to have smaller social support networks due to age-related losses of immediate family/friends while experiencing declining health and increasing mobility limitations (Kemperman et al., 2019). A study found that local social ties (nearly 60% of them being non-kin) are frequently accessed and highly embedded in older adults’ networks, especially among socially disadvantaged older adults (Cornwell & Goldman, 2021).

Studies have also found significant cross-sectional and longitudinal associations between higher perceived NSC and lower social isolation and loneliness in older adults, though these associations tend to be weaker among Black and Hispanic older adults (Choi, 2024; Massihzadegan & Stokes, 2023; Qin et al., 2024; Stephens & Phillips, 2022; Tang et al., 2024; Taylor et al., 2023; Yang & Moorman, 2021). The negative impacts of social isolation and loneliness, representing both objective and subjective experiences of social disconnection, on health and longevity in later life are substantial, for example, a 50% increase in the risk of developing dementia, a 30% increased risk of incident coronary artery disease or stroke, and a 26% increased risk of all-cause mortality (National Academies, 2020). A lack of strong social support networks, social isolation, and loneliness, particularly among older adults living alone, are also significant risk factors for adverse mental health outcomes (Domènech-Abella et al., 2019). Conversely, strong neighborhood networks play a crucial role in mitigating poor mental health outcomes among vulnerable older adults during crises such as the COVID-19 pandemic (Gan & Best, 2021).

Research has also shown that NSC is influenced by structural characteristics of a neighborhood, such as socioeconomic burden and associated neighborhood physical disorder (NPD) (Carpiano, 2007). Greater neighborhood concentrated disadvantage—characterized by low income and education, high unemployment, and female-headed households—is linked to the loss of both kin and non-kin network members over time (Goldman et al., 2023). NPD, generally defined by features such as trash, vacant buildings, and crime (Ross & Mirowsky, 2001), is significantly associated with lower physical activity, poorer physiological health, social isolation, and loneliness in older adults (Qin et al., 2024). Especially among those with physical, cognitive, or sensory vulnerabilities, poor physical infrastructure associated with NPD presents direct barriers to safe movement, increasing the risk of falls, anxiety, and depressive mood. NPD can also heighten perceptions of neighborhood unsafety and crime risk. All these can lead older adults to perceive lower NSC and limit outdoor mobility, indicating the importance of considering NPD, along with social support networks, when examining NSC and mobility in older adults.

Although the relationship between NSC and health has been extensively studied, little research has been done on how homebound state is associated with NSC among U.S. older adults. An estimated 5% to 10% of older adults in the U.S. are homebound, typically defined as rarely or never leaving the home due to chronic medical conditions, functional or cognitive impairments, and/or mental health issues such as depression and anxiety (Ornstein et al., 2020; Xiang et al., 2020; Yang et al., 2021). Compared to their non-homebound counterparts, homebound older adults experience significantly greater social isolation and loneliness (Cudjoe et al., 2022; Ezeokonkwo et al., 2021; Yang et al., 2021), as well as more frequent use of healthcare services, particularly emergency department visits and hospitalizations (Mitsutake et al., 2021; Oseroff et al., 2023). A seven-year study of Medicare beneficiaries also revealed that homebound state fluctuates: while some older adults remained homebound, transitioned to nursing homes, or died, others regained independence, though many later became homebound again (Ankuda et al., 2021). Further research is needed to examine how NSC may be associated with homebound state, including its progression and recovery.

This study utilized data from the 2022 and 2023 U.S. National Health and Aging Trends Study (NHATS) to examine the associations between NSC and homebound status among older adults. Our analysis was guided by the neighborhood-based theory of social capital for health (Carpiano, 2006, 2007). The theory posits that, independently of the factors (e.g., social support network, NPD, sociodemographic and health status) that shape it, NSC is a foundation of social capital and contributes, through connectedness and trust, to the residential quality of life and, ultimately, health among residents of the community/neighborhood. Conversely, a lack of NSC may lead to diminished social control and a reduced sense of neighborhood safety, which can discourage older adults, especially those with cognitive, physical, or mental health challenges, from leaving their homes.

Study hypotheses were: (H1) higher levels of NSC would be significantly associated with a lower likelihood of homebound state (outcome) in 2022; and (H2) favorable changes in NSC between 2022 and 2023 would be significantly associated with a higher likelihood of recovery from the homebound state (homebound in 2022 to non-homebound in 2023), but unfavorable changes in NSC would be significantly associated with a higher likelihood of continued or newly homebound state in 2023, controlling for sociodemographic and health characteristics, social support network size, and NPD. The findings of this study provide added insights into the homebound state in older adults, whose numbers are increasing, by examining the role of NSC that extends beyond individual health problems.

## Methods

### Data Source

NHATS collects data annually from a nationally representative panel of Medicare beneficiaries (age 65+) about their physical, functional, cognitive, and sensory capacity, social, physical, and technological environments, and participation in valued activities. Since its inception in 2011, NHATS has added the first, second, and third replenishment samples in 2015, 2022, and 2023, respectively (https://www.nhats.org/). The 2022 and 2023 NHATS data were collected in in-person interviews. In this study, we first focused on 5,593 sample persons in 2022 who lived in their own homes or residential care communities (but not in nursing homes) and self-reported data (i.e., no proxy interview). Second, we focused on 4,907 sample persons who provided self-reported data on the homebound states and other variables in both 2022 and 2023. We excluded proxy-interviewed sample persons (*n* = 241 in 2022 and *n* = 390 in 2023) to ensure that all data were self-reported. The analysis of de-identified public-use data in this study was exempt from the authors’ institutional review board review.

### Measures

#### Homebound versus Non-Homebound Older Adults

NHATS respondents were asked, “In the last month, how often did you leave your building/home to go outside?” Response categories were never, rarely (once a week or less), some days (2–4 days a week), most days (5-6 days a week), and every day (7 days a week). Following previous NHATS-based studies (Ornstein et al., 2020; Xiang et al., 2020), we defined homebound older adults as those who never/rarely (≤ once a week) left home in the past month as opposed to non-homebound older adults who went out at least 2 days a week. Those who were not homebound were divided into two groups—those who went out 2–4 days a week and those who went out 5+ days a week—for more nuanced analysis.

#### Change in Homebound State, 2022-2023

Of the sample persons who were interviewed in both 2022 and 2023, we compared their going-out frequencies in 2022 and 2023 and created the change variable with the following attributes: (1) non-homebound in 2022 and 2023; (2) homebound in 2022 but not homebound in 2023 (no longer homebound); and (3) not homebound in 2022 but homebound in 2023 (newly homebound) or homebound in both 2022 and 2023 (continued to be homebound).

#### Neighborhood Social Cohesion (NSC) and Changes Between 2022 and 2023

NSC was measured with the total score from three items—people in this community know each other very well, are willing to help each other, and can be trusted—each on a 3-point scale (0 = *do not agree*, 1 = *agree a little*, and 2 = *agree a lot*), drawn from the social cohesion scale originally developed by Sampson (1997). “Community” in NHATS was defined as “the community where the sample person lives.” In previous NHATS-based studies (e.g., Qin et al., 2024), the community was equated to the sample person’s neighborhood. Unweighted Cronbach’s alphas for the 3-item scale were .75 in 2022 and .76 in 2023. We calculated 2022-2023 changes based on score differences between the two years: no change, decreased (=unfavorable change), and increased (=favorable change).

#### Sociodemographic Factors in 2022 and 2023

These included age (65–74 [reference category], 75–84, 85+); gender (female vs. male); race/ethnicity (non-Hispanic White [reference category], non-Hispanic Black, Hispanic, all other); residential type (care community vs. own home); living arrangement (living alone vs. living with spouse and/or other); and family income (<$43,000, $43,000-$83,999 [reference category], $84,000+, and missing).

#### Cognitive health in 2022 and 2023

was measured using the NHATS’ dementia classification (no dementia [reference category], possible dementia, and probable dementia), which was based on: (1) doctor diagnosis of dementia or Alzheimer’s disease (yes or no); and (2) scores from cognitive tests for three domains: memory, orientation, and executive function (Kasper et al., 2024). A possible dementia classification was assigned when the person scored <1.5 SD below the mean in one domain of the cognitive tests. A probable dementia classification was assigned when the person was diagnosed with dementia or scored <1.5 standard deviations (SD) below the mean in at least two domains of the cognitive tests.

#### Physical/functional health in 2022 and 2023

was measured with (a) the number of diagnosed chronic medical conditions (0-8: arthritis, cancer, hypertension, heart disease, stroke, diabetes, lung disease, osteoporosis); (b) activity-limiting chronic pain (yes or no); (c) activity-limiting fall worry (yes or no); and (d) use of any mobility assistive devices (cane, walker, wheelchair, or scooter) outside the home (yes or no). We report percentages of those who needed help to go outside and the past-year incidents of hospitalization for descriptive purposes only.

#### Mental health indicator in 2022 and 2023

was depression/anxiety symptoms in the past month, assessed with the Patient Health Questionnaire-4 (PHQ-4) (Kroenke et al., 2009). The PHQ-4 includes the first two items (PHQ-2; loss of interest and depressed mood) from the 9-item PHQ-9 for depression (Kroenke et al., 2003) and the first two items (GAD-2; nervous/anxious feeling and inability to control worrying) from the 7-item Generalized Anxiety Disorder Scale (Spitzer et al., 2006). Each item was scored on a 4-point scale (0 = not at all; 1 = several days; 2 = more than half the days; 3 = nearly every day), with the total score ranging from 0 to 12. Unweighted Cronbach’s alphas for the PHQ-4 for the study sample were .75 in 2022 and .77 in 2023.

#### Social Support Network Size in 2022 and 2023

Social support network size was defined as the number of people (up to 5) the sample person talked to “about important things in life, including good or bad things that happened and problems or concerns.” This did not differentiate between kin and non-kin networks.

#### Neighborhood Physical Disorder (NPD) in 2022 and 2023

NPD was measured using three items that the interviewer reported on the sample person’s residential area conditions—littered glasses on some sidewalk streets, graffiti on building walls, and vacant houses or stores. Each item was rated as none (i.e., no indication of disorder), a little, some, a lot, and missing/could not observe. In this study, we used the following categories: (1) “none” for all three items, (2) “a little” if any item received such a rating, (3) “some/a lot” if any item received “some” of “a lot” rating; and (4) “missing/could not observe” for all three items.

### Analysis

All analyses were conducted with Stata/MP 19.5’s svy function (College Station, TX) to account for NHATS’s stratified, multistage sampling design. All estimates presented in this study are weighted except for sample sizes. First, we used *χ*^2^ and *t* tests to compare the characteristics of homebound older adults versus those who went out 2–4 days a week and those who went out 5+ days a week in 2022. Second, we also used *χ*^2^ and *t* tests to compare the characteristics of those who were no longer homebound (i.e., recovery) in 2023, the newly or continued homebound in 2023, and those who remained non-homebound in 2022 and 2023. Third, we fitted two logistic regression models to test H1 (associations between NSC and homebound state, compared to 2–4 days and 5+ days of outings, respectively, in 2022). Fourth, we fitted a multinomial logistic regression model to test H2 (associations between 2022-2023 changes in NSC and changes in homebound state), with those who remained non-homebound in both years as the reference group. The variance inflation factor (VIF) diagnostics, using a cut-off of 2.50 (Allison, 2012), from linear regression models indicated that multicollinearity among covariates was not a concern. Logistic regression results are presented as adjusted odds ratios (aORs) with 95% confidence intervals (CI), and multinomial logistic regression results are presented as relative risk ratios (RRR) with 95% CI. Significance was set at *p <* .05.

## Results

### Characteristics of Homebound Older Adults in 2022

Table 1 shows that in 2022, 4.9% (95% CI = 4.2%-5.6%) of the study population were homebound in the past month, 11.6% went outside 2–4 days a week, and the rest went outside 5+ days a week. Compared to both groups of non-homebound older adults, homebound older adults included a higher proportion of women and had more cognitive, physical/functional, and mental health problems. Compared to those who went out 5+ days a week, homebound older adults reported significantly fewer people in their social support network and lower perceived NSC, and a higher proportion of them lived in areas with NPD. Homebound older adults did not significantly differ from those who went out 2–4 days a week with respect to social support network size and NSC, but lived in a neighborhood with more NPD.

**Table 1.** Characteristics of homebound older adults compared to non-homebound peers in 2022

	Homebound (1)	Went out 2-4 days a week (2)	Went out 5+ days a week (3)	*p* between (1) & (2)	*p between (1) & (3)*
N	416	873	4,304		
% of all (95% CI)	4.9 (4.2-5.6)	11.0 (10.0-12.2)	84.1 (82.9-85.4)		
Age group (years, %)				.213	<.001
65-74	40.3	46.9	59.2		
75-84	38.0	35.8	32.6		
85+	21.7	17.3	8.2		
Female (%)	73.6	64.4	52.5	.003	<.001
Race/ethnicity (%)				.231	<.001
Non-Hispanic White	60.8	59.6	81.8		
Non-Hispanic Black	8.1	16.6	7.3		
Hispanic	11.6	15.8	6.5		
Other	5.3	8.1	4.4		
Living alone (%)	42.3	34.2	26.5	.049	<.001
Care community residence (%)	11.6	9.1	2.8	.315	<.001
Total income (%)				.052	<.001
<$43,000	65.4	58.4	34.8		
$43,000-$83,999	11.6	16.5	26.2		
$84,000+	8.3	14.2	28.1		
Missing	14.7	10.9	10.9		
Dementia status (%)				<.001	<.001
No dementia	66.4	81.0	90.8		
Possible dementia	17.7	9.2	6.0		
Probable dementia	15.9	9.8	3.2		
No. of chronic medical conditions (M, SE)	3.25 (0.16)	2.86 (0.07)	2.22 (0.03)	.002	<.001
Activity-limiting pain (%)	60.9	48.8	28.5	.002	<.001
Activity-limiting fall worry (%)	35.2	23.4	8.1	.002	<.001
Use of mobility assistive device outside (%)	54.2	43.8	11.9	.002	<.001
Need help to go out (%)	46.2	26.0	3.7	<.001	<.001
Any hospitalization in the past year (%)	26.3	29.9	13.1	.855	<.001
Depression/anxiety symptoms, M (SE)	3.39 (0.16)	2.55 (0.12)	1.53 (0.05)	<.001	<.001
Social support network size, M (SE)	1.91 (0.09)	1.98 (0.07)	2.33 (0.04)	.534	<.001
NPD (%)				.046	<.001
None	78.5	83.5	87.2		
A little	10.5	10.6	7.5		
Some/a lot	9.0	3.9	2.8		
Missing/could not observe	2.0	2.0	2.5		
NSC, M (SE)	3.28 (0.12)	3.58 (0.09)	4.23 (0.04)	.059	<.001

NPD = Neighborhood physical disorder; NSC = Neighborhood social cohesion.

*Note.* Probability values were calculated based on Pearson’s *χ*^2^ tests and *t*-tests.

### Characteristics of the No-Longer Homebound and the Newly and Continued Homebound in 2023

Table 2 shows that of those who self-reported data in both 2022 and 2023, 2.2% (*n* = 154) were homebound in 2022 but no longer homebound in 2023, and 4.3% remained homebound (*n* = 162) or became homebound (*n* = 169) in 2023, and 93.5% remained non-homebound in both years. Of the 416 homebound sample persons in 2022, 85 were not included in this analysis due to reported death (*n* = 37), relocation to a nursing home (*n* = 5) or an assisted living facility but not interviewed (*n* = 4), or proxy-interview or other non-interview status (*n* = 39) in 2023.

**Table 2.** Characteristics of older adults in 2023 by changes in homebound state between 2022 and 2023

	Homebound in 2022, but not homebound in 2023 (1)	Homebound in both 2022 and 2023 or became homebound in 2023 (2)	Not homebound in 2022 and 2023 (3)	*p* between (1) & (2)	*p* between (1) & (3)	*p* between (2) & (3)
N (%)	154 (2.2)	331 (4.3)	4,461 (93.5)			
Age group (years, %)				.745	.037	<.001
65-74	41.2	42.4	47.0			
75-84	37.0	32.8	41.1			
85+	21.8	24.8	11.6			
Female (%)	77.1	68.4	54.9	.186	<.001	.033
Race/ethnicity (%)				.179	<.001	<.001
Non-Hispanic White	65.6	62.5	82.1			
Non-Hispanic Black	15.9	15.4	8.4			
Hispanic	12.9	19.6	4.5			
Other	5.6	2.5	5.0			
Living alone (%)	39.5	41.0	29.2	.883	.179	.004
Care community residence (%)	12.4	12.3	4.0	.983	.002	<.001
Total income (%)				.574	<.001	<.001
<$43,000	59.5	62.5	34.0			
$43,000-$83,999	12.8	11.7	24.9			
$84,000+	9.1	5.1	30.4			
Missing	18.6	20.7	10.7			
Dementia status (%)				.243	<.001	<.001
No dementia	72.5	64.6	89.3			
Possible dementia	16.4	16.7	5.9			
Probable dementia	18.1	18.7	4.8			
No. of chronic medical conditions, M (SE)	3.02 (01.3)	3.32 (0.11)	2.29 (0.03)	.125	<.001	<.001
Activity-limiting pain (%)	45.6	55.3	30.8	.197	.017	<.001
Activity-limiting fall worry (%)	28.9	34.6	9.6	.379	<.001	<.001
Mobility aid use outside (%)	53.9	51.1	15.9	.678	<.001	<.001
Need help to go out (%)	37.5	62.2	5.5	<.001	<.001	<.001
Any hospitalization in the past year (%)	29.3	37.4	15.8	.641	<.001	<.001
Depression/anxiety symptoms, M (SE)	3.20 (0.22)	3.93 (0.28)	1.49 (0.04)	.142	<.001	<.001
Social support network size, M (SE)	2.39 (0.10)	1.78 (0.11)	2.40 (0.04)	<.001	.864	<.001
NPD (%)				.213	<.001	.003
None	73.7	80.6	89.4			
A little/some/a lot	19.2	12.5	6.5			
Some/a lot	7.1	5.3	2.4			
Missing/could not observe	0	1.5	1.7			
NSC, 2022-2023 change (%)				.034	.011	.026
No change	32.8	33.5	42.3			
Decreased	22.2	37.0	27.4			
Increased	44.9	29.5	30.3			

NPD = Neighborhood physical disorder; NSC = Neighborhood social cohesion.

*Note.* Probability values were calculated based on Pearson’s *χ*^2^ tests and *t-*tests.

Those who were no longer homebound and those who were newly homebound or continued to be homebound did not significantly differ from each other on sociodemographic and most health characteristics. Compared to those who were non-homebound in both years, these two groups were older and included higher proportions of women, racial/ethnic minorities, and those who lived in care communities, had more health problems, and lived in neighborhoods with more NPD. However, those who were no longer homebound in 2023 did not differ from those who remained non-homebound in terms of living arrangement and social support network size. Of the three groups, those who were no longer homebound in 2023 included the highest proportion reporting favorable changes in NSC (44.9% vs. 30.3% among the non-homebound group and 29.5% among the continued or newly homebound group), and those who were newly or continued to be homebound included the highest proportion reporting unfavorable changes in NSC.

### Associations Between NSC and Homebound State: Logistic Regression Results

Table 3 shows that the higher NSC scores were associated with significantly lower odds of homebound state (aOR = 0.92, 95% CI = 0.84-0.99 when compared to the 2–4 times weekly outings; aOR = 0.79, 95% CI = 0.73-0.86 when compared to the 5+ times outings). These findings support H1. Of the covariates, only possible dementia was associated with higher odds of homebound state, compared to the 2–4 times outings. Compared to the 5+ times outings, age 85+, female gender, being Black or Hispanic, care community residence, low income (<$43,000), possible and probable dementia, and most health indicators were associated with higher odds of homebound state, but a greater social support network size was associated with lower odds.

**Table 3.** Association between NSC and homebound state (never/rarely went out in the past month) in 2022 Results from logistic regression

	Homebound vs. Went out 2–4 days a week aOR (95%CI)	Homebound vs. Went out 5+ days a week aOR (95% CI)
NSC	0.92 (0.84-0.99)*	0.79 (0.73-0.86)***
Age group: vs. 65-74		
75-84	1.22 (0.81-1.84)	1.46 (0.93-2.29)
85+	1.24 (0.67-2.29)	2.19 (1.25-3.81)**
Female vs. Male	1.31 (0.94-1.82)	1.95 (1.42-2.67)***
Race/ethnicity: vs. Non-Hispanic White		
Non-Hispanic Black	0.77 (0.45-1.32)	1.96 (1.18-3.28)*
Hispanic	1.07 (0.61-1.88)	3.35 (1.98-5.65)***
Other	0.63 (0.35-1.13)	1.52 (0.71-3.23)
Living alone vs. Not living alone	1.40 (0.96-2.04)	0.97 (0.66-1.41)
Care community residence vs. Own home	1.10 (0.62-1.96)	3.14 (1.61-6.12)**
Total income vs. $43,000-$83,999		
<$43,000	1.27 (0.71-2.28)	1.92 (1.09-3.38)*
$84,000+	1.06 (0.45-2.46)	1.08 (0.52-2.21)
Missing	1.81 (0.89-3.67)	1.56 (0.77-3.16)
Dementia status vs. No dementia		
Possible dementia	2.11 (1.11-4.01)*	1.93 (1.09-3.43)*
Probable dementia	1.68 (0.93-3.05)	2.77 (1.64-4.69)***
No. of medical conditions	1.10 (0.97-1.25)	1.12 (0.99-1.27)
Activity-limiting pain vs. No activity-limiting pain	1.25 (0.83-1.88)	1.88 (1.31-2.69)**
Activity-limiting fall worry vs. No activity-limiting fall worry	1.46 (0.93-2.29)	2.41 (1.59-3.64)***
Mobility aid use	1.08 (0.75-1.56)	3.13 (2.16-4.53)***
Depression/anxiety symptoms	1.05 (0.98-1.13)	1.09 (1.03-1.15)**
Social support network size	0.94 (0.82-1.08)	0.85 (0.73-0.98)*
NPD: vs. None		
A little	0.85 (0.53-1.35)	0.83 (0.52-1.32)
Some/a lot	2.70 (0.97-7.53)	2.23 (0.83-5.97)
Missing/could not observe	1.17 (0.51-2.68)	0.50 (0.21-1.15)
Model statistics	*N* = 1,289; Population *N* = 7.8 million; design df = 56; F (23,34) = 3.29; *p* < .001	*N* = 4,720 Population *N* = 43.9 million; design df = 56; F (23,34) = 26.02; *p* < .001

NSC = Neighborhood social cohesion; NPD = Neighborhood physical disorder.

*p < .05; **p < .01; ***p < .001.

### Associations Between Changes in NSC and Changes in Homebound State: Multinomial Logistic Regression Results

The first column of Table 4 shows that a favorable change in NSC was associated with higher odds of recovery from the homebound state in 2023 (RRR = 1.63, 95% CI = 1.01- 2.62). This finding partially supports H2. Of the covariates, female gender, Hispanic ethnicity, possible dementia, mobility aid use, higher depressive/anxiety symptoms, and “a little” NPD were positively associated with recovery from the homebound state. The second column of Table 4 shows that the changes in NSC were not a significant factor. Being Black or Hispanic, low-income or missing income, possible and probable dementia, more medical conditions, fall worry, mobility aid use, and higher depressive/anxiety symptoms were associated with higher odds of newly or continued homebound state, but a greater social support network size was associated with lower odds (aOR = 0.73, 95% CI = 0.59-0.91).

**Table 4.** Associations between changes in NSC and changes in homebound state, 2022-2023: Results from multinomial logistic regression

	No longer homebound in 2023 vs. Not homebound in 2022 & 2023 RRR (95%CI)	Continued to be or newly homebound in 2023 vs. Not homebound in 2022 & 2023 RRR (95%CI)
Changes in NSC, 2022-2023: vs. No change		
Decreased	0.82 (0.46-1.46)	1.24 (0.77-2.00)
Increased	1.63 (1.01-2.62)*	1.02 (0.67-1.55)
Age group: vs. 65-74		
75-84	0.73 (0.37-1.45)	0.64 (0.40-1.02)
85+	0.84 (0.42-1.66)	1.12 (0.61-2.05)
Female vs. Male	2.12 (1.28-3.51)**	1.29 (0.79-2.12)
Race/ethnicity: vs. Non-Hispanic White		
Non-Hispanic Black	1.33 (0.79-2.24)	1.52 (1.04-2.22)*
Hispanic	2.26 (1.18-4.33)*	3.75 (2.14-6.59)***
Other	1.30 (0.43-3.87)	0.43 (0.18-1.02)
Living alone vs. Not living alone	0.88 (0.42-1.87)	1.02 (0.69-1.51)
Care community residence vs. Own home	2.12 (0.89-5.02)	1.85 (0.85-4.03)
Total income vs. $43,000-$83,999		
<$43,000	2.00 (0.85-4.74)	2.07 (1.03-4.18)*
$84,000+	0.73 (0.22-2.42)	0.54 (0.19-1.57)
Missing	2.31 (0.82-6.46)	2.38 (1.08-5.26)*
Dementia status vs. No dementia		
Possible dementia	2.29 (1.18-4.43)*	2.12 (1.22-3.69)**
Probable dementia	1.32 (0.59-2.95)	2.13 (1.17-3.88)*
No. of medical conditions	1.13 (0.94-1.35)	1.29 (1.12-1.48)**
Activity-limiting pain vs. No activity-limiting pain	0.75 (0.37-1.51)	1.07 (0.73-1.57)
Activity-limiting fall worry vs. No activity-limiting fall worry	1.34 (0.66-2.73)	1.67 (1.07-2.63)*
Mobility aid use	3.54 (2.19-5.71)***	2.25 (1.43-3.54)**
Depression/anxiety symptoms	1.16 (1.05-1.29)**	1.21 (1.11-1.31)***
Social support network size in 2023	1.10 (0.97-1.24)	0.73 (0.59-0.91)**
NPD in 2023^a^: vs. None		
A little	2.26 (1.30-3.92)**	1.04 (0.62-1.75)
Some/a lot	1.95 (0.77-4.91)	0.91 (0.29-2.89)
Model statistics	*N* = 4,838; Population *N* = 24.2 million; design df = 56; F (46,11) = 8.51; *p* < .001	

NSC = Neighborhood social cohesion; NPD = Neighborhood physical disorder.

^a^Those with missing data (*N* = 70 [63 for non-homebound in 2022 and 2023, 0 for no longer homebound in 2023, and 7 for continued homebound or newly homebound in 2023]) were excluded from the analysis.

*p < .05; **p < .01; ***p < .001

## Discussion

We examined the associations between perceived NSC and homebound state in U.S. older adults. In 2022, about 5% of Medicare beneficiaries age 65 and older were homebound, that is, never/rarely went outside in the past month. Even after adjusting for sociodemographic and health statuses, social support network size, and NPD, higher perceived NSC was significantly associated with lower odds of homebound state. Our study also shows the dynamic nature of homebound states, consistent with prior research (Ankuda et al., 2021; Xiang et al., 2020). Between 2022 and 2023, similar proportions of the study population remained homebound, became homebound, or recovered from being homebound. Notably, improvements in perceived NSC over this period were significantly associated with higher odds of recovery from homebound status. While changes in NSC were not significantly related to remaining or becoming homebound, a larger social support network was associated with lower odds of these outcomes.

The relationship between NSC and homebound status may be bidirectional. Lower levels of NSC may increase the likelihood of becoming homebound, while being homebound can, in turn, diminish perceived NSC. Older adults who perceive their neighbors as unhelpful, unfriendly, or untrustworthy may be less inclined to leave their homes due to safety concerns or a lack of motivation for social engagement. An unwelcoming neighborhood environment may also discourage them from seeking support from local social ties, further reinforcing their homebound status. At the same time, homebound older adults have limited opportunities for spontaneous or meaningful interactions with neighbors, which can erode their sense of connection and trust in the community. This lack of engagement may contribute to social disconnectedness, which, in turn, increases the risk of social isolation, loneliness, and depression/anxiety (Santini et al., 2020), further shaping negative neighborhood perceptions. Prior research links social isolation to homebound progression among both Black and white older adults and among those without dementia at baseline (Cudjoe et al., 2022; Yang et al., 2021). However, given the observational nature of this study, causal inferences cannot be drawn.

We also propose two possible explanations for the significant association between favorable changes in NSC and the higher odds of recovery from a homebound state. First, increased social interactions, an essential ingredient of favorable NSC, may have followed recovery, such as after overcoming an illness. Alternatively, neighborhood-based support may have been mobilized in response to disability onset, enabling older adults to leave their homes more frequently (e.g., attending religious services). While NHATS primarily defines social network members as providers of emotional support, they may also facilitate outdoor mobility through instrumental or informational support (e.g., transportation assistance and resources for independent living). Supporting this perspective, a study of older adults in a rural Midwestern city found that having more companionship-providing network members was associated with greater environmental mastery, stronger social connections, and positive social influence, including encouragement to engage in healthy behaviors (Ashida et al., 2018).

The observed association between larger social support network size and lower odds of remaining or becoming homebound may be interpreted similarly. While we did not distinguish between kin and non-kin network members in our analyses, prior research suggests that stronger perceived neighborhood social ties and interactions are associated with higher rates of adding non-kin members and lower rates of adding kin members over time (Goldman et al., 2023).

Our findings also indicate that recovery from a homebound state was positively associated with living in neighborhoods characterized by “a little” NPD and with Hispanic ethnicity. While NPD is often interpreted as a marker of social deterioration, reduced safety, and weakened social control, all factors that may discourage outdoor activity (Robinette et al., 2016), this association may be more complex. Taylor et al. (2023) found that among Black older adults, higher levels of NPD were associated with lower levels of social isolation from friends. The authors noted that an objective measure of NPD may not accurately reflect residents’ subjective perceptions, particularly among Black and Hispanic older adults, and speculated that NPD may reflect higher neighborhood population density and tightly knit communities with strong social bonds.

Although Hispanic ethnicity was positively associated with recovery from a homebound state, both Black and Hispanic older adults had significantly higher odds of being homebound, as well as of remaining or becoming homebound over time, even after controlling for other sociodemographic and health-related factors. These racial/ethnic disparities are concerning. Because the 2022 and 2023 NHATS data were collected during the COVID-19 pandemic, which disproportionately negatively affected Black and Hispanic older adults (Garcia et al., 2021), these older adults may also have been staying home even more than during the pre-pandemic times.

Our findings on the significant association of homebound state and the continuation of and transition into homebound state with dementia, chronic illnesses, pain, fall worry, and mobility aid use align with previous research. Xiang et al. (2020) identified probable dementia and the use of a walker or wheelchair as significant risk factors for both the onset and continuation of a homebound state. Yang et al. (2021) found that pain increased the risk of homebound progression, regardless of dementia status. Similarly, Choi et al. (2020) demonstrated that, even after accounting for fall incidents and changes in health status, fall worry limited both informal and formal social engagement over time, suggesting its role as a contributing factor to becoming homebound.

Interestingly, recovery from a homebound state the following year, compared to non-homebound state in both years, was also significantly associated with possible dementia, mobility aid use, and higher depressive/anxiety symptoms. This likely reflects the poorer baseline health status among homebound older adults and suggests that even after recovery, they may continue to require assistance to leave their homes. The elevated depressive and anxiety symptoms observed in this group may, in part, stem from feelings of dependence and helplessness. Older adults who rely on others for outdoor mobility may continue to experience these psychological burdens, contributing to the persistence of depressive and anxiety symptoms even after regaining some mobility.

Overall, our findings suggest that higher levels of NSC may help protect older adults from becoming homebound and could foster social engagement and facilitate recovery from homebound status. Those with cognitive, physical, or functional health problems often rely on family, friends, or neighbors for assistance. When family and friends are unavailable, knowing and trusting neighbors can facilitate safer and easier interactions and help-seeking behaviors. According to the neighborhood-based theory of social capital for health, high perceived NSC fosters trust and social connectedness, enhancing older adults’ sense of safety, increasing community engagement, and improving well-being.

This study has several limitations. First, the sample size for the longitudinal analysis of homebound transitions was small, and the findings from observational data are correlational rather than causal. Second, the NHATS used abbreviated NSC, NPD, and social support network size measures. More robust measures for these constructs are needed. Third, the 2022 and 2023 NHATS data were collected during the COVID-19 pandemic, whose lingering effects likely influenced older adults’ frequency of going out, NSC, social support networks, and psychological well-being. Further research is needed to explore the relationships between homebound status and neighborhood and social network characteristics, including the directionality of these relationships.

Despite these limitations, our findings have important implications for interventions aimed at increasing neighborhood social capital to better address the health and social needs of the increasing number of homebound older adults. Community/neighborhood-based programming is essential to facilitate older adults’ engagement in meaningful social and educational activities. The findings from the previous reviews of evidence-based interventions for reducing social isolation and loneliness among older adults (Gardiner et al., 2018; Paquet et al., 2023) can offer some programming ideas. These include developing infrastructure for social prescription and establishing neighborhood-based support groups with educational and recreational components, particularly in communities where racial/ethnic minority older adults are concentrated. Senior centers, many of which serve minoritized older adults, can serve as focal points for engaging homebound older adults by offering diverse health-promotion, educational, recreational, and volunteer opportunities, along with nutritious meals and multilingual services.

In conclusion, this study provides empirical evidence that NSC plays a protective role against the onset of homebound status and facilitates recovery among older adults. These findings highlight the critical importance of neighborhood social capital in supporting health and mobility in later life. In the context of a rapidly aging population, strategic investments in neighborhood-based physical and social health programs are essential, particularly for racial/ethnic minority older adults, who are disproportionately affected by homebound status and face greater barriers to recovery.

## Data Availability

The data used in this study (the National Aging and Health Trends Study) are in public domain.*
